# Rethinking Pulmonary Embolism Management with an Interventional Perspective

**DOI:** 10.3390/jcm14093085

**Published:** 2025-04-29

**Authors:** Panayotis K. Vlachakis, Stergios Soulaidopoulos, Emmanouil Mantzouranis, Panagiotis Theofilis, Paschalis Karakasis, Anastasios Apostolos, Ioannis Kachrimanidis, Maria Drakopoulou, Costas Tsioufis, Konstantinos Toutouzas

**Affiliations:** 1 1st Department of Cardiology, Hippokration General Hospital, National and Kapodistrian University of Athens, 11527 Athens, Greece; soulaidopoulos@gmail.com (S.S.); mantzoup@gmail.com (E.M.); panos.theofilis@hotmail.com (P.T.); anastasisapostolos@gmail.com (A.A.); iskachrimanidis@gmail.com (I.K.); mdrakopoulou@hotmail.com (M.D.); ktsioufis@gmail.com (C.T.); ktoutouz@gmail.com (K.T.); 2 Second Department of Cardiology, Hippokration General Hospital, Aristotle University of Thessaloniki, 54642 Thessaloniki, Greece; pakar15@hotmail.com

**Keywords:** pulmonary embolism, management, risk-stratification, interventional therapies, thrombolysis

## Abstract

Pulmonary embolism (PE) remains a major cardiovascular emergency associated with significant morbidity and mortality. Despite advances in risk stratification models, accurately predicting which intermediate-high-risk patients will deteriorate remains challenging. Systemic thrombolysis, while effective in high-risk PE, is not a viable option for a significant proportion of patients due to contraindications, and its efficacy in the intermediate-high-risk group remains inconclusive. Drawing parallels from acute myocardial infarction and stroke, where percutaneous interventions have revolutionized treatment, interventional therapies are emerging as a promising alternative for PE management. However, challenges persist regarding optimal patient selection, procedural timing, and balancing efficacy with safety. The establishment of pulmonary embolism response teams (PERTs) has played a crucial role in streamlining decision-making and facilitating access to advanced therapies. As novel catheter-based techniques continue to evolve, the field of PE management is undergoing a paradigm shift, mirroring the transformation seen in acute coronary and cerebrovascular care, positioning interventional approaches at the forefront of therapy.

## 1. Introduction

Among the various manifestations of cardiovascular disease, pulmonary embolism (PE) remains a prevalent and life-threatening condition that continues to constitute diagnostic and therapeutic challenges for modern healthcare clinicians. Despite significant efforts and resources dedicated to medical research, PE is the third most common cardiovascular cause of death globally, and its incidence rates have shown a slight upward trend over time [1].

The clinical severity, especially the presence of hemodynamic instability upon admission, may result in mortality rates of up to 30% within the initial 30 days for patients with acute PE [2]. Additionally, approximately 30% of survivors may later experience recurrent venous thromboembolism or encounter chronic disabling symptoms with potential life-threatening consequences [3]. Data revealed that PE accounts for approximately 300,000 annual deaths in Europe, while in the United States, national health statistics estimate around 110,000 PE-related deaths each year, with no significant decline in age-adjusted mortality rates over time [4,5]. Although the absolute number is lower than in Europe, the burden in the United States remains substantial and has shown no significant improvement in age-adjusted mortality rates over recent years [6]. Moreover, PE imposes a significant economic burden, covering expenses related to hospitalization, diagnostics, anticoagulation treatment, and ongoing complication management. This results in an estimated healthcare cost of EUR 3.8 billion in Europe and USD 18.9 billion in the United States annually [7].

As mentioned earlier, even after successful treatment, PE survivors remain at risk for recurrence, chronic thromboembolic disease and chronic thromboembolic pulmonary hypertension (CTEPH), leading to functional impairment and increased mortality [3]. Preventing these complications requires early diagnosis, precise risk stratification, and optimal management. While anticoagulation is the mainstay of treatment, systemic thrombolysis (ST) remains the standard for high-risk PE and rescue therapy for deteriorating patients [8]. However, its use is limited by a significant bleeding risk, contraindications, and variable efficacy, highlighting the need for alternative therapeutic options [9].

Following the paradigm of acute myocardial infarction and stroke, where interventional options have taken the lead and demonstrated superior outcomes, there is growing interest in catheter-based therapies for PE. These interventional approaches offer targeted clot removal with potentially lower bleeding risks, ranging from local thrombolysis with reduced-dose fibrinolytics to mechanical aspiration thrombectomy and direct clot retrieval techniques [10,11]. With high procedural success rates and a favorable safety profile, these strategies may redefine the treatment landscape for PE, particularly in patients at intermediate-to-high risk [12].

This review aims to explore the evolving landscape of PE management, with a particular focus on interventional strategies. By examining the current evidence, clinical implications, and future directions of catheter-based treatments, we aim to highlight their potential role in reshaping the standard of care for PE.

## 2. Risk Stratification and Current Treatment Paradigm

Risk stratification in acute PE is essential for guiding therapeutic decisions and determining prognosis. Current classification systems categorize patients based on hemodynamic stability, imaging findings, and biomarker levels to estimate the risk of adverse outcomes. While PE severity likely exists on a continuum rather than discrete categories, risk models are employed to facilitate clinical decision-making.

The American Heart Association (AHA) and European Society of Cardiology (ESC) have both established risk classification models. The AHA broadly classifies PE as low-risk, submassive (intermediate-risk), or massive (high-risk). In contrast, the ESC provides a more granular approach by further subdividing intermediate-risk patients into intermediate-low and intermediate-high categories [8,13]. Intermediate-high-risk patients exhibit right ventricular (RV) dysfunction and elevated cardiac troponin, placing them at an increased risk of early decompensation [8]. Additionally, tools such as the pulmonary embolism severity index (PESI) and its simplified version (sPESI) are widely adopted to assess short-term mortality risk, with higher scores indicating worse prognosis (Table 1) [14,15]. However, even with these stratification frameworks, accurately identifying intermediate-high-risk patients who will deteriorate remains an ongoing challenge.

No single clinical, laboratory, or imaging parameter reliably predicts clinical deterioration in intermediate-high-risk PE patients [16]. Traditional risk models incorporate a mix of static and dynamic variables, but newer approaches suggest that repeated clinical assessments, such as those using the National Early Warning Score (NEWS), may provide superior predictive value [3]. Ongoing trials like Higher-Risk Pulmonary Thrombolysis (HI-PEITHO) are currently evaluating the role of NEWS in early detection of decompensation [3]. Additionally, markers such as serum lactate and cardiac troponin have been linked to short-term mortality risk, particularly when combined with RV dysfunction on echocardiography [17,18]. However, the interpretation of these biomarkers must be approached with caution, as variability in assay sensitivity and specificity, as well as confounding factors like renal dysfunction, age, and comorbidities, may limit their diagnostic accuracy and consistency across clinical settings. Emerging data suggest that the normalization of systolic blood pressure, heart rate, brain natriuretic peptide (BNP), and troponin within 48 h may help identify patients with a lower risk of deterioration [2].

Emerging risk assessment strategies integrate echocardiographic parameters, such as RV/LV ratio, tricuspid annular plane systolic excursion (TAPSE), and systolic excursion velocity, to improve prognostication [19]. Additionally, computed tomography (CT) assessments, including 3D reconstruction and dual-energy CT, hold promise for the more precise evaluation of clot burden and distal vasculature perfusion, though their routine use remains limited due to concerns about radiation exposure and contrast administration [20].

Treatment strategies for intermediate-high-risk PE patients remain a subject of debate. While anticoagulation is the cornerstone of therapy, systemic thrombolysis (ST) remains reserved for those with high-risk PE or signs of hemodynamic deterioration [8]. However, systemic thrombolysis carries a high risk of major bleeding, making it unsuitable for some patients. The choice between unfractionated heparin (UFH) and low-molecular-weight heparin (LMWH) is also crucial, as studies suggest that a substantial proportion of PE patients treated with standard UFH regimens remain subtherapeutic in the first 48 h, potentially increasing the risk of recurrent events [21].

Despite the utility of structured risk models, individualized patient assessment remains essential. Pre-existing conditions such as chronic pulmonary hypertension, cardiomyopathy, or renal disease can complicate the interpretation of RV dysfunction and biomarker elevations, potentially leading to misclassification of PE severity. In these cases, considering the location of the emboli and estimated clot burden is crucial to ensure that clinical and imaging findings accurately reflect the impact of PE rather than underlying comorbidities.

## 3. Systemic Thrombolysis: A Double-Edge Sword

The management of individuals with PE adheres to four fundamental principles: restoring proper blood flow, maintaining hemodynamic stability, promoting tissue oxygenation, and preventing the recurrence of the condition [16]. ST remains the primary reperfusion strategy for patients with high-risk PE due to its potential to rapidly dissolve thrombi, restore pulmonary perfusion, and improve hemodynamic stability [8,13,22]. Studies have demonstrated that ST reduces total and PE-related mortality compared to anticoagulation alone, with a significant reduction in PE recurrence [22,23]. Given the urgency of intervention in hemodynamically unstable patients, the widespread availability of intravenous thrombolytic agents allows for prompt reperfusion with minimal delay. However, despite being the recommended first-line therapy for high-risk PE, only a small percentage of eligible patients receive ST due to concerns regarding bleeding risk [24]. Data suggest that over half of patients with high-risk PE do not receive thrombolysis due to a perceived increased risk of bleeding, contributing to persistently high mortality rates [25].

The major limitation of ST is its high bleeding risk, including major extracranial hemorrhage and intracranial hemorrhage (ICH), which occurs in approximately 10% of treated patients [9]. In unstable patients, this bleeding risk is further compounded by mechanisms such as venous congestion, right atrial hypertension, and paradoxical embolization via a patent foramen ovale, increasing the likelihood of hemorrhagic complications [26]. Meta-analyses indicate that while ST reduces mortality in unstable patients (number needed to treat = 59), it also substantially increases major bleeding risk (number needed to harm = 18), including a significant risk of ICH (number needed to harm = 78) [27]. The concern over these bleeding complications has limited the use of ST, with data suggesting an overall utilization rate of only 2.5%, despite guideline recommendations [28].

Attempts to mitigate bleeding risk through reduced-dose thrombolysis have been explored, as seen in the Moderate Pulmonary Embolism Treated with Thrombolysis (MOPETT) trial, which demonstrated that low-dose thrombolysis reduced pulmonary hypertension and shortened hospital stay. However, it did not significantly impact mortality or long-term functional outcomes and its findings are limited by a small sample size and inconsistent inclusion criteria [29].

The role of ST in intermediate-high-risk PE remains controversial, as current evidence fails to demonstrate a clear mortality benefit. The Fibrinolysis for Patients with Intermediate-Risk Pulmonary Embolism (PEITHO) trial, one of the largest randomized controlled trials assessing thrombolysis in intermediate-risk PE, found that while tenecteplase reduced the risk of hemodynamic decompensation (2.6% vs. 5.6%), this benefit came at the cost of significantly increased major bleeding (6.3% vs. 1.2%) and hemorrhagic stroke [23]. Furthermore, long-term follow-up from the PEITHO trial found no significant difference in mortality, persistent RV dysfunction, or functional impairment between patients who received ST and those who received anticoagulation alone [30]. The ongoing Pulmonary Embolism International Trial (PEITHO)—3 trial seeks to better define the role of reduced-dose thrombolysis versus standard anticoagulation in intermediate-high-risk PE (NCT04430569). Additionally, meta-analyses from 2023 suggest a possible short-term benefit of thrombolysis in this population, though the overall evidence remains weak [31].

## 4. Interventional Approaches for PE Management

**Figure 1:**
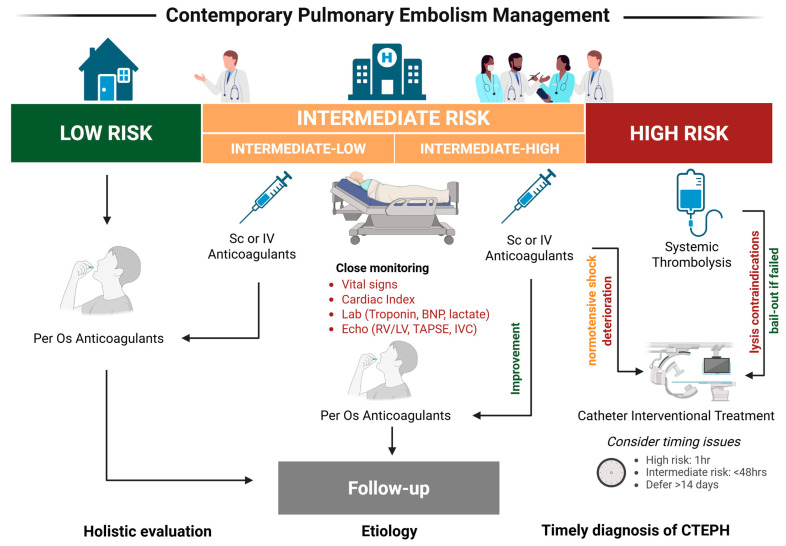
Contemporary management of pulmonary embolism in the interventional era. Created with BioRender.com (accessed on 2 April 2025). Abbreviations: *BNP*: brain natriuretic peptide; *CTEPH*: chronic thormboembolic pulmonary hypertension; *echo*: echocardiogram; *IVC*: inferior vena cava; *IV*: intravenously; *RV/LV*: right ventricle/left ventricle ratio; *Sc*: subcutaneously; *TAPSE:* tricuspid annular plane systolic excursion.

It was in 1924, even before the use of ST, when Martin Kischner performed the first successful emergency surgical embolectomy on a patient with PE [32]. A century later, in the rapidly evolving landscape of interventional cardiology, we have witnessed the introduction of numerous innovative interventional therapeutic approaches that are currently the subject of scientific and clinical scrutiny. These approaches seek to lower the occurrence of hemodynamic collapse while minimizing the risk of bleeding. In line with the 2019 ESC guidelines on acute PE diagnosis and management, the consideration of interventional, catheter-directed treatments is recommended for patients experiencing intermediate to high-risk PE in case of treatment failure, defined as hemodynamic deterioration despite anticoagulation. Additionally, interventional management is recommended for patients with high-risk PE when ST has proven either ineffective or is contraindicated (class IIa-Level of evidence C) [8]. Figure 1 illustrates the evolving paradigm of PE management, emphasizing the integration of interventional strategies alongside conventional therapies to optimize patient outcomes across different risk categories.

Presently accessible catheter-based reperfusion methods encompass catheter-directed thrombolysis (CDT) and percutaneous mechanical embolectomy (PBM).

### 4.1. Catheter-Directed Therapy (CDT)

Catheter-directed therapy (CDT) represents a targeted interventional approach for PE management, aiming to enhance thrombus resolution while minimizing systemic thrombolytic exposure. This technique involves the use of specialized infusion catheters to deliver thrombolytic agents directly into the thrombus, reducing the risk of major bleeding associated with ST. CDT can be performed with standard infusion catheters or enhanced by mechanical disruption techniques such as ultrasound-assisted thrombolysis (USAT) [33].

The standard approach to CDT involves placing an infusion catheter within the pulmonary arteries, followed by a controlled administration of thrombolytics, typically alteplase, at lower doses than those used in systemic thrombolysis. While drug regimens may vary, typical protocols involve an initial bolus (4–6 mg) followed by a continuous infusion at 0.5–1 mg/h per catheter for 12–24 h, with the total dose not exceeding 30 mg [34]. Mechanical enhancement of CDT, particularly with USAT, has gained attention as a means to improve drug penetration and accelerate thrombus dissolution. The EkoSonic™ Endovascular System (Boston Scientific) is an example of a USAT device that combines ultrasound energy with local thrombolytic infusion [35]. This system utilizes a filament equipped with multiple ultrasound transducers that emit pulsed, high-frequency, low-energy ultrasound waves, which help disaggregate fibrin structure, increasing the exposure of thrombus-bound plasminogen activators and enhancing fibrinolysis [36]. While USAT may offer increased efficiency compared to standard CDT, it is associated with higher procedural costs [37]. Specifically, time-driven activity-based costing analyses estimate the total cost of USAT at approximately USD 9017 per procedure—more than double the cost of standard CDT (USD 3889)—with substantial contributions from device-related materials and intensive care utilization [38].

Several clinical studies have assessed the efficacy and safety of CDT in patients with intermediate-high-risk PE. The Ultrasound Accelerated Thrombolysis of Pulmonary Embolism (ULTIMA) trial demonstrated that USAT resulted in a significant 22% reduction in RV/LV diameter ratio at 24 h, while standard anticoagulation alone failed to produce meaningful changes [39]. Similarly, the Submassive and Massive Pulmonary Embolism Treatment With Ultrasound Accelerated Thrombolysis Therapy (SEATTLE II) study, which included 150 patients, reported a 27% reduction in RV/LV ratio at 48 h, with a major bleeding rate of less than 1% [40]. The Catheter-Directed Thrombolysis vs. Anticoagulation in Patients with Acute Intermediate-High-Risk Pulmonary Embolism (CANARY) trial attempted to compare USAT with systemic thrombolysis over a three-month follow-up period but was terminated early due to the COVID-19 pandemic. Although the study did not meet its primary endpoint, a composite analysis suggested a potential reduction in mortality and persistent RV dysfunction in the intervention arm [41]. The Recombinant tPA by Endovascular Administration for the Treatment of Submassive Pulmonary Embolism Using Pharmaco-mechanical Catheter Directed Thrombolysis for the redUction of Thrombus burdEn (RESCUE) trial, which evaluated the hybrid use of CDT with the Bashir catheter, reported a 33% reduction in RV/LV ratio at 48 h with a similarly low major bleeding rate [42]. Meta-analyses incorporating both randomized controlled trials and observational studies suggest that CDT may be associated with lower 30-day and in-hospital mortality compared to anticoagulation alone, without significantly increasing major bleeding risk [43,44]. However, robust randomized data comparing CDT to anticoagulation in terms of long-term clinical outcomes remain limited.

### 4.2. Percutaneous Mechanical Embolectomy (PME)

Percutaneous mechanical embolectomy (PME) is an interventional technique that mechanically removes thromboembolic material from the pulmonary arteries. Unlike CDT, PME can be performed without the use of thrombolytic agents, offering a potential advantage in patients with contraindications to systemic fibrinolysis or an elevated bleeding risk. Various mechanical thrombectomy devices are available, utilizing different mechanisms to fragment, aspirate, or disrupt thrombi to restore pulmonary circulation [37].

Several methods exist within PME, including thrombus fragmentation, rotational thrombectomy, rheolytic thrombectomy, and aspiration embolectomy [37]. Thrombus fragmentation is the most basic technique, historically performed using a pigtail catheter that rotates manually around a guidewire to break apart the clot [45]. This approach, while cost-effective and widely available, has largely been supplanted by more advanced devices due to its limited efficacy and potential for distal embolization [13,46]. Rotational thrombectomy systems, such as those incorporating sinusoidal rotating tips with active aspiration, offer improved clot removal by preventing distal embolization, though they carry a risk of endothelial injury [47,48]. Rheolytic thrombectomy, exemplified by the AngioJet system (Boston Scientific), employs high-pressure saline jets to disrupt thrombi while simultaneously aspirating debris [49]. However, this method has been associated with complications such as hemolysis-induced bradyarrhythmia, prompting an FDA warning regarding its use in PE [45,50].

Aspiration embolectomy has emerged as a leading PME approach, leveraging large-bore catheters to directly extract thrombi while minimizing reliance on thrombolytics. Several dedicated aspiration systems have been developed, including the FlowTriever (Inari Medical) and Indigo (Penumbra) systems [51]. The FlowTriever device uses a dual mechanism of clot displacement via expandable nitinol mesh disks followed by aspiration with large-bore catheters [37]. The Indigo system, by contrast, relies on continuous suction generated by an electronic pump, with an optional separator wire to assist in thrombus disintegration [52]. Unlike FlowTriever, which incorporates larger-profile catheters for rapid thrombus retrieval, Indigo’s smaller and more flexible catheters allow for access to more distal clot burdens.

Clinical studies have evaluated the safety and efficacy of PME in intermediate-high-risk PE. The Evaluating the Safety and Efficacy of the Indigo aspiration system in Acute Pulmonary Embolism (EXTRACT-PE) study, which investigated suction thrombectomy using the Indigo system in 119 patients, reported a mean reduction in the RV/LV diameter ratio of 0.43 (27.3%), with a 30-day all-cause mortality of 2.5% and a major bleeding rate of 1.7% [52]. Similarly, the FlowTriever Pulmonary Embolectomy (FLARE) study assessed mechanical thrombectomy with the FlowTriever system in 106 patients, demonstrating a 0.38 (25.1%) reduction in RV/LV ratio, with a 30-day mortality of 1% and a major bleeding rate of 1% [51]. While FLARE was a single-arm study, when compared to a parallel context arm in which patients received non-FlowTriever therapies, the in-hospital mortality was 1.9% in the thrombectomy group compared to 29.5% in the context arm [51]. The FlowTriever All-Corner Registry for Patient Safety and Hemodynamics (FLASH) registry further analyzed FlowTriever outcomes in 799 patients, reporting a 6-month mortality of 4.6%, a 35.0% reduction in RV/LV ratio, and a low prevalence of CTEPH (1.0%) [53].

While these findings suggest that PME provides effective clot burden reduction with low mortality and bleeding rates, randomized data comparing PME to anticoagulation alone are currently lacking. The ongoing Randomized Controlled Trial of Large-Bore Thrombectomy Versus Anticoagulation In Intermediate-Risk Pulmonary Embolism (PEERLESS II) trial, a randomized study evaluating PME versus anticoagulation in intermediate-high-risk PE patients with additional risk factors, aims to clarify the role of thrombectomy in this setting (NCT06055920) [54]. Until such data are available, PME remains an evolving intervention, best suited for patients in whom thrombolysis is contraindicated or rapid hemodynamic stabilization is required.

## 5. Timing of Intervention: Fighting Against Time

A challenging question revolves around determining the optimal timing for reperfusion in acute PE cases classified as high-risk for deterioration under standard treatment. A growing body of evidence suggests that a time-sensitive approach to reperfusion is associated with improved short-term outcomes, particularly in patients with high-risk pulmonary embolism.

Indeed, the administration of systemic thrombolytic therapy within 24 h after symptom onset has been linked to a reduced need for inotropic and respiratory support compared to delayed thrombolysis, which has been associated with a substantially higher risk of both in-hospital and long-term mortality [55]. Similarly, a significant decline in the efficacy of thrombolysis in reducing 30-day cardiovascular mortality has been observed when administered later than 8.5 h after symptom onset, independent of bleeding events [56]. From a pathophysiological standpoint, this clinical benefit of early thrombolysis likely reflects the rapid reduction in pulmonary vascular resistance and RV overload due to the prompt restoration of pulmonary arterial blood flow, ultimately improving hemodynamics and gas exchange [57]. Furthermore, considering the long-term sequelae of PE, earlier reperfusion appears to reduce the prevalence of functional limitations and the risk of developing CTEPH.

When considering percutaneous mechanical interventions, the proper timing of intervention is equally critical. While high-risk patients necessitate immediate intervention, intermediate-risk patients may tolerate a short delay. However, studies have shown that early interventions—within 24 to 48 h of presentation—are more effective and safer than delayed interventions performed beyond 48 h [58]. Current evidence suggests this 24–48 window applies equally to CD and PME for intermediate-high-risk PE, though some controversy exists regarding PME’s potential suitability for more organized clots in later presentations. Reflecting this urgency, a recent ESC protocol recommends initiating treatment for high-risk patients within 60 min of diagnosis, or up to 90 min if the diagnosis is made in a facility without access to percutaneous intervention [59]. During these interventions, continuous monitoring of vital parameters, including oxygen saturation, systemic arterial and pulmonary pressures, and heart rhythm, is essential to ensure hemodynamic stabilization [60]. Additionally, intraprocedural imaging plays a crucial role in localizing thrombi and assessing embolic burden, thereby optimizing procedural success.

Despite these insights, systemic thrombolysis remains underutilized, largely due to concerns regarding hemorrhagic complications. Notably, data indicate that two out of three patients with pulmonary embolism and hemodynamic instability do not receive any form of reperfusion treatment [61]. Given these disparities, interventional strategies, akin to acute myocardial ischemia management, ensure more complete and timely recanalization for restoring distal flow, particularly in cases where bleeding concerns delay thrombolytic therapy. These approaches may ultimately contribute to improved long-term clinical outcomes.

## 6. Challenges and Practical Considerations in Interventional PE Management

The decision to perform percutaneous intervention for PE and the choice of technique remain challenging due to the heterogeneity in patient characteristics, clinical presentation, and available interventional strategies. Currently, there is no universally accepted algorithm for selecting the optimal approach, with the decision largely relying on institutional protocols, physician expertise, and individual patient factors [34]. Clinical data suggest that catheter-directed treatments, assuming adequate operator expertise, can achieve hemodynamic stabilization with in-hospital survival rates approaching 90%. However, the safety and efficacy of these techniques vary based on patient risk stratification, comorbidities such as chronic kidney disease, and bleeding risk [33].

Successful implementation of interventional PE treatments depends heavily on operator experience and access to advanced technology. CDT is generally considered a safe technique, but periprocedural complications, particularly bleeding events, remain a concern, with extracranial major bleeding occurring in 1–9.2% of cases and intracranial hemorrhage in approximately 1% [13,34]. Mechanical thrombectomy, while offering rapid clot debulking, carries additional risks due to larger catheters and potential trauma to pulmonary structures [62]. Moreover, thrombus fragmentation during PME can lead to distal embolization, increasing right ventricular afterload and worsening hemodynamic status. The need for skilled operators, specialized training, and real-time imaging further complicates widespread adoption, particularly in nontertiary care centers [33,34].

Logistical challenges play a critical role in determining the feasibility of interventional PE management. The need for rapid diagnosis, immediate team mobilization, and access to specialized equipment often limits availability, particularly in community hospitals or non-PCI-capable centers [58]. ESC guidelines recommend initiating treatment within 60 min of diagnosis, or up to 90 min if the facility lacks percutaneous intervention capabilities [59]. Despite these recommendations, real-world implementation is frequently hindered by systemic delays, emphasizing the importance of improved hospital coordination, streamlined protocols, and dedicated PE response teams [58]. To address these barriers, the incorporation of telemedicine consultations and the establishment of regional referral networks may facilitate timely decision-making and expand access to specialized care. Additionally, continuous monitoring during the procedure—including vital signs, oxygenation, and systemic and pulmonary arterial pressures—is essential to ensure hemodynamic stability, further necessitating a well-organized infrastructure.

The comparative efficacy and safety of CDT and PME remain areas of active investigation. While CDT has demonstrated favorable hemodynamic outcomes with a lower bleeding risk compared to full-dose thrombolysis, the need for prolonged thrombolytic infusion raises concerns about delayed reperfusion [37]. Conversely, PME offers a thrombolytic-free option that may be particularly advantageous in patients with contraindications to systemic anticoagulation [37]. However, mechanical techniques vary widely in their mechanisms and risk profiles. Rheolytic thrombectomy, for example, has been associated with higher rates of bradyarrhythmia and hemolysis, while aspiration techniques require large-bore access, potentially increasing vascular complications [45,50]. Table 2 provides a summary of the safety outcomes across various interventional strategies for PE management.

Several ongoing trials aim to address these uncertainties (Table 3). These studies will be crucial in refining patient selection criteria and optimizing treatment protocols for interventional PE management.

## 7. The Role of Pulmonary Embolism Response Teams (PERTs)

Pulmonary embolism response teams (PERTs) have become an essential component in the multidisciplinary management of intermediate- and high-risk PE, streamlining rapid assessment and optimizing treatment strategies. These teams integrate specialists from various disciplines, including cardiology, pulmonology, intensive care, vascular medicine, hematology, emergency medicine, interventional radiology, and cardiothoracic surgery, to ensure a comprehensive and individualized approach. Evidence from multiple institutions supports the benefits of PERT implementation, with studies demonstrating reductions in ICU and overall hospital length of stay, decreased time to therapeutic anticoagulation, and lower rates of major bleeding and inferior vena cava filter placement [66]. Data from the Cleveland Clinic showed an 8.7% absolute reduction in major bleeding and a 3.8% reduction in 30-day mortality, while international studies have reported up to a 9.1% decrease in PE-related mortality [67]. However, outcomes have varied across institutions, with some centers reporting no significant mortality benefit but an increased use of risk stratification tools and more judicious application of advanced therapies [68,69]. Additionally, PERTs contribute to medical education by enhancing clinician confidence in managing PE, particularly regarding thrombolysis and surgical embolectomy [70]. Recognized in the latest ESC guidelines with a class IIa recommendation, PERTs continue to evolve, offering a structured yet adaptable framework to improve decision-making and patient outcomes in complex PE cases [8].

## 8. Conclusions

The management of PE has entered a transformative era, driven by advances in interventional strategies and a growing emphasis on multidisciplinary collaboration. While randomized controlled trials remain the gold standard for evaluating medical therapies, the rapid evolution of catheter-based treatments has outpaced the availability of high-level evidence. Despite this, real-world data and observational studies continue to support the efficacy and safety of percutaneous interventions, leading to their increasing adoption in clinical practice. PERTs have further refined patient selection and individualized treatment, ensuring optimal outcomes. As new trials seek to define the precise role of interventional therapies, the field of PE management is shifting away from systemic thrombolysis toward safer and more targeted approaches. As innovation, rigorous evaluation, and the integration of advanced technologies enhance patient care while minimizing risks, the cardiology community will witness a significant shift toward interventional management, ultimately redefining the standard of care in acute PE treatment.

## Figures and Tables

**Table 1:** American Heart Association and European Society of Cardiology risk stratification models for pulmonary embolism.

Risk Category	European Society of Cardiology	American Heart Association	Clinical Criteria
Low-Risk	Hemodynamically stable, without signs of RV strain or myocardial injury	Hemodynamically stable, with normal cardiac biomarkers and no RV dysfunction	No imaging or biomarker evidence of strain; ESC recommends using PESI I–II or sPESI = 0 to support classification
Intermediate-Low	Stable, with **either** RV dysfunction **or** elevated biomarkers (not both)	Hemodynamically stable with **either** RV dysfunction, **or** elevated cardiac biomarkers, **or both**. *(The AHA does not subcategorize this group into low- or high-risk*)	ESC supports classification using PESI class III–IV or sPESI ≥ 1; clinical monitoring is advised
Intermediate-High	Stable, but with **both** RV dysfunction and elevated cardiac biomarkers	Hemodynamically stable with **either** RV dysfunction, **or** elevated cardiac biomarkers, **or both**. *(The AHA does not subcategorize this group into low- or high-risk*.)	Includes evidence of myocardial stress (e.g., ↑ troponin, ↑ BNP) and RV strain (e.g., RV/LV ratio ≥ 1.0 or TAPSE < 16 mm); ESC supports classification using PESI class III–IV or sPESI ≥ 1
High	Hemodynamic compromise: sustained hypotension, cardiac arrest, or bradycardia	Same as ESC	Immediate risk of mortality; urgent reperfusion strategies usually indicated

Abbreviations: *BNP*: brain natriuretic peptide; *BP*: blood pressure; *CT*: computed tomography; *echo*: echocardiogram; *ESC*: European Society of Cardiology; *LV*: left ventricle; *PESI*: pulmonary embolism severity index; *RV*: right ventricle; *sPESI*: simplified pulmonary embolism severity index; *TAPSE*: tricuspid annular plane systolic excursion.

**Table 2:** Safety profiles of interventional pulmonary embolism therapies.

Trial	Device	Population and Sample Size	Intervention	Follow-Up	Safety
ULTIMA (2013) [39]	EkoSonic	Intermediate-high-risk PE (n = 59)	Anticoagulation + USAT (tPA 10mg	90 days	No deaths or major bleeds; 3 minor bleeds
SEATTLE II (2015) [40]	EkoSonic	Intermediate-high-risk PE (n = 150)	Anticoagulation + USAT (tPA 12–24 mg)	30 days	7 deaths, 15 major bleeds
OPTALYSE PE (2018) [36]	EkoSonic	Intermediate-risk PE (n = 101)	Anticoagulation + USAT (tPA 4–12 mg across 4 regimens)	365 days	5 major bleeds within 72 h
SUNSET sPE (2021) [63]	EkoSonic vs. Cragg-McNamara	Intermediate-risk PE (n = 81)	Anticoagulation + USAT (tPA 4–8 mg) vs. standard CDT	90 days	2 major bleeds, 3 minor bleeds, 1 in-hospital death
CANARY (2022) [41]	Cragg-McNamara	Intermediate-high-risk PE (n = 94)	Anticoagulation + CDT (tPA 12–24 mg)	90 days	1 BARC Type 3a major bleed
RESCUE (2022) [42]	BASHIR	Intermediate-risk PE (n = 109)	Anticoagulation + CDT (tPA 7–14 mg)	30 days	1 death, 3 major bleeds
Kroupa et al. (2022) [64]	Cragg-McNamara	Intermediate-high-risk PE (n = 23)	Anticoagulation + CDT (tPA 20 mg)	30 days	No BARC Type 3c or 5 major bleeds
FLARE (2019) [51]	FlowTriever	Intermediate-risk PE (n = 106)	Anticoagulation + FlowTriever	30 days	0 deaths; major bleeding 0.9% at 48 h
EXTRACT-PE (2021) [52]	Indigo	Intermediate-risk PE (n = 119)	Anticoagulation + Indigo	30 days	1.1% mortality; 1.6% major bleeding at 48 h
FLAME (2023) [65]	FlowTriever	High-risk PE (n = 104)	Anticoagulation + FlowTriever vs. other therapies	In hospital	Death: 1.9% vs. 29.5%; Major bleeding: 11.3% vs. 24.6%

Abbreviations: *BARC*: Bleeding Academic Research Consortium; *CDT*: catheter-directed thrombolysis; *PE*: pulmonary embolism; *tPA*: tissue plasminogen activator; *USAT*: ultrasound-assisted thrombolysis.

**Table 3:** Overview of ongoing clinical trials evaluating interventional treatments for pulmonary embolism.

Trial	Design	Intervention	Primary Outcome	Sample Size	Estimated Completion Date
PRAGUE-26 (NCT05493163)	Open-label, Phase 4	CDT vs. anticoagulation in intermediate-high-risk pts	All-cause death, hemodynamic instability, recurrent PE	558	01-2028
HI-PEITHO (NCT04790370)	Single-blind, Phase 4	USAT vs. anticoagulation in intermediate-high-risk pts	All-cause death, hemodynamic instability, recurrent PE	544	08-2026
PE-TRACT (NCT05591118)	Open-label, Phase 3	CDT vs. anticoagulation in Intermediate-high-risk pts	PVO2, NYHA classification, incidence of major bleeding	500	01-2028
PEERLESS II (NCT06055920)	Open-label	PME vs. anticoagulation in intermediate-high-risk pts	Clinical deterioration, all-cause hospital readmission, bailout therapy, dyspnea	1200	07-2026
TORPEDO-NL (NCT06833827)	Open-label	CDT vs. ST in high-risk pts	Composite incidence of all-cause mortality, treatment failure, major bleeding, and all-cause stroke	111	01-2029
ESCADlys-PE (NCT06487052)	Open-label	CDT vs. anticoagulation in intermediate-high-risk pts	A decrease in the ratio of RV/LV diameters	100	12-2027
STORM-PE (NCT05684796)	Open-label	PME vs. anticoagulation in intermediate-high-risk pts	Change in RV/LV ratio	100	10-2026
PERSEVERE (NCT06588634)	Open-label	CDT vs. standard of care in high-risk pts	Composite clinical endpoint of all-cause mortality, cardiac arrest, bailout therapy, major bleeding, ECMO life support	200	08-2027
CATCH-PE II (NCT06672081)	Open-label	CIT vs. standard of care in high-risk pts	Composite endpoint of mortality (all-cause) and recurrent cardiac arrest or persistent/recurrent shock	315	06-2027

Abbreviations: *CDT*: catheter-directed thrombolysis; *CIE*: catheter-interventional treatment; *NYHA*: New York Heart Association; *PVO2*: peak oxygen consumption; *pts*: patients; *PME*: percutaneous mechanical embolectomy; *PE*: pulmonary embolism; *ST*: systemic thrombolysis; *USAT*: ultrasound-assisted thrombolysis.

## Data Availability

Not applicable.
